# Cardiovascular magnetic resonance patterns of biopsy proven cardiac involvement in systemic sclerosis

**DOI:** 10.1186/s12968-016-0289-3

**Published:** 2016-10-21

**Authors:** Patrick Krumm, Karin A. L. Mueller, Karin Klingel, Ulrich Kramer, Marius S. Horger, Tanja Zitzelsberger, Reinhard Kandolf, Meinrad Gawaz, Konstantin Nikolaou, Bernhard D. Klumpp, Joerg C. Henes

**Affiliations:** 1 Department of Radiology, Diagnostic and Interventional Radiology, University of Tübingen, Hoppe-Seyler-Str. 3, Tübingen, 72076 Germany; 2 Department of Internal Medicine III, Cardiology and Cardiovascular Medicine, University of Tübingen, Tübingen, Germany; 3 Department of Pathology and Neuropathology, Molecular Pathology, University of Tübingen, Tübingen, Germany; 4 Department of Internal Medicine II, Rheumatology, University of Tübingen, Tübingen, Germany

**Keywords:** Systemic sclerosis, Cardiomyopathy, Rheumatic heart disease, Cardiovascular magnetic resonance

## Abstract

**Background:**

To determine morphological and functional cardiovascular magnetic resonance (CMR) patterns in histopathologically confirmed myocardial involvement in patients with systemic sclerosis (SSc).

**Methods:**

Twenty patients (6 females; mean age 41 ± 11 years) with histopathologically proven cardiac involvement in SSc in the years 2008–2016 were retrospectively evaluated. Morphological, functional and late gadolinium enhancement (LGE) images were acquired in standard angulations at 1.5 T CMR. Pathologies were categorized: 1) Pericardial effusion; 2) pathologic left (LV) or right ventricular (RV) contractility (hypokinesia, dyssynchrony, and diastolic restriction); 3) reduced left (LV-EF) and right ventricular ejection fraction (RV-EF); 4) fibrosis and/or inflammation (positive LGE); 5) RV dilatation. 95 % confidence intervals (CI) were calculated for appearance of pathologic EF and RV dilatation.

**Results:**

Seven patients (35 %) had positive CMR findings in three categories, 9 patients (45 %) in four categories and 4 patients (20 %) in five categories. The distribution of pathologic findings was: minimal pericardial effusion in 7 patients (35 %), moderate pericardial effusion >5 mm in nine patients (45 %); abnormal LV or RV contractility in 19 patients (95 %), reduced LV or RV function in 14 patients (70 %; 95 % CI: 51–88 %), pathologic LGE in all patients, RV dilatation in 6 patients (30 %; 95 % CI: 15–54 %).

**Conclusions:**

CMR diagnosis of myocardial involvement in SSc requires increased attention to subtle findings. Pathologic findings in at least three of five categories indicate myocardial involvement in SSc.

**Electronic supplementary material:**

The online version of this article (doi:10.1186/s12968-016-0289-3) contains supplementary material, which is available to authorized users.

## Background

Systemic sclerosis (SSc) is a rare chronic systemic autoimmune disease associated with different autoantibodies, the most common are anti-topoisomerase I (Anti-Scl-70) and anti-centromere antibodies (ACA). SSc is associated with high morbidity and mortality [1]. The pathogenesis of SSc is not entirely understood but microvascular changes are suspected to be the main cause of connective tissue infiltration and fibrosis [2]. Besides cutaneous manifestations, visceral involvement especially of bowel, lung, heart and kidney is more common and occurs earlier in patients with diffuse cutaneous subset of SSc (dcSSc) than in patients with limited cutaneous SSc (lcSSc) [3, 4]. In general, the prognosis in patients with myocardial involvement is poor [2].

In these patients with cardiac involvement, non-segmental perfusion defects can be observed in myocardial stress perfusion cardiovascular magnetic resonance (CMR), suggesting resemblances in the pathogenesis of SSc associated Raynaud’s phenomenon and transient myocardial ischemia [5]. Cardiac Raynaud’s phenomenon may be one reason for subsequent myocardial fibrosis, in addition to non-specific hypertrophy.

Several CMR studies have pointed out characteristic findings in patients with SSc. These are: Pericardial effusion [6], left ventricular (LV) and right ventricular (RV) diastolic and systolic dysfunction, LV hypertrophy, perfusion defects and myocardial late gadolinium enhancement (LGE) [5, 7–10].

Standard treatment of SSc includes cyclophosphamide, which can be cardiotoxic [11]. In our patient group with severe scleroderma, CMR was part of a routine diagnostic work up in the course of preparation and risk stratification for autologous stem cell transplantation and escalation of potentially cardiotoxic therapy. Myocardial histopathological fibrosis and inflammation in SSc is by trend associated with cardiac events [12].

Aim of this study was to systematically determine morphological and functional CMR imaging patterns in systemic sclerosis in patients with histopathologically confirmed myocardial involvement in SSc.

## Methods

### Study population

Twenty patients (6 female, 14 male; mean age 40.7 ± 10.8 years; range [19; 56] years) with a history of SSc for 3.0 years (±2.9 years; range [0.5; 10] years) were retrospectively included. One patient had lcSSc, 19 had dcSSc. The mean modified Rodnan skin score was 26 (±11; range [4; 45]). Troponin I was elevated in 13 patients (65 %), brain natriuretic peptide in 6 patients (30 %). Anti-Scl-70 antibodies were positive in 15 patients (75 %), additionally anti-centromere antibodies were positive in two patients (10 %). Three patients (15 %) had pulmonary arterial hypertension, seven patients (35 %) had pathologic Holter ECG.

This study retrospectively evaluated a subpopulation of patients with cardiac involvement in SSc as proven by endomyocardial biopsy (EMB) previously undergoing CMR in the years 2008–2016. Clinical findings in a part of the study group (16 patients) were published by Mueller et al. [12]. All patients were clinically diagnosed by an experienced rheumatologist according to the 2013 criteria of the American College of Rheumatology [13]. None of the patients presented with symptoms of cardiac failure. For clinical and laboratory suspicion of myocardial involvement, patients underwent EMB including histopathologic, immunohistologic and molecular pathological evaluation at median 14 days after CMR.

### Endomyocardial biopsy and analysis

EMB was sampled from the right ventricular septum. Histopathological and immunohistological analyses were performed by experienced cardiopathologists as described by Mueller et. al. [12]: The degree of fibrosis was rated in percent of the biopsy area. Inflammation was graded 0–4: grade 0 = no inflammation; 1 = single inflammatory cells; 2 = few foci of inflammation; grade 3 = several foci of inflammation; grade 4 = pronounced inflammation. Molecular detection of viral genomes was performed.

### CMR acquisition

CMR was performed on a 1.5 T scanner (Magnetom Avanto, Siemens Healthcare, Erlangen, Germany) equipped with a gradient system with a maximum strength of 45 mT/m and a maximum slew rate of 200 T/m/s. A six channel body array coil and six coils of the spine coil were used for reception of MR-signals. All images were acquired in breath hold technique with ECG triggering.

For functional imaging, Steady State Free Precession (SSFP) cine loops (repetition time (TR) 3 ms; echo time (TE) 1.5 ms; flip angle 60°, slice thickness 5 mm; matrix 256x192; 25 frames per cardiac cycle) were acquired in standard angulations: four-chamber view (4CV), two-chamber view (2CV) and a stack of short-axis slices (gap 5 mm) covering both entire ventricles from base to apex. For anatomical overview, a T2w Half-fourier Acquisition Single-shot Turbo Spin-Echo (HASTE) in 5 mm gapless axial slices covering the thorax was performed. Optimal inversion time (TI) was determined with an inversion time localizer (TI Scout) SSFP, magnetization preparation: slice selective inversion pulse, 20 ms increment for inversion recovery measurement, (TR 24 ms, TE 1.12 ms, flip angle 60°, slice thickness 8 mm). The inversion time localizer was acquired 9 min after administration of 0.15 mmol Gadobutrol per kg body weight (Gadovist, Bayer Healthcare, Leverkusen, Germany) in a mid-cavity short-axis slice. LGE imaging was performed 10–15 min after contrast agent application with a 2D T1-weighted inversion recovery spoiled gradient echo sequence (segmented k-space readout, TR 8.0 ms, TE 4.9, TI as predetermined and incrementally adapted, flip angle 30°, slice thickness 8 mm) in standard angulations with corresponding slice positions to the functional imaging sequences. For all sequences, the field of view was 280–340 mm adapted to patient’s size, kept to a minimum, respectively.

### Image analysis

Image analysis for the retrospective study analysis was performed by two independent readers (eleven and five years of experience in cardiac imaging), aware of the histopathologic biopsy result, using an offline workstation CVI42 (Circle Cardiovascular Imaging, Calgary AB, Canada). Initial reading of the images in clinical routine had been performed by different readers, blinded to the result of the subsequent biopsy. The results of the initial reports were evaluated categorically in ‘cardiac SSc involvement’ and ‘no cardiac SSc involvement’.

#### Morphology and function

Contractility was evaluated according to the following criteria: Presence of hypokinesia, dyskinesia, intraventricular synchrony, and diastolic function for both ventricles respectively, in a visual qualitative analysis.

Functional parameters were determined by volumetry of the left and right ventricle in a stack of short-axis slices according to the modified Simpson rule [14]. End-diastole and end-systole were determined manually. Endocardial contours were drawn with help of a semi-automated region growing algorithm in both ventricles. Epicardial contours of the left ventricle were drawn manually. Left ventricular end-diastolic volume (LV-EDV) with the corresponding left ventricular end-diastolic volume index (LV-EDVI) normalized to body surface area (BSA), left ventricular ejection fraction (LV-EF) as well as left ventricular myocardial mass (LV-MM) and the respective index (LV-MMI) were calculated. Accordingly, right ventricular end-diastolic volume (RV-EDV), right ventricular end-diastolic volume index (RV-EDVI) and right ventricular ejection fraction (RV-EF) were assessed. Ventricular dilatation was determined according to the recently published criteria by Kawel-Boehm et al. 2015 [15]. Ventricles were classified as dilated if the ventricular end-diastolic volume index was above the upper limit calculated as mean +2 standard deviations (SD) of the normal collective regarding gender and age <60 years: LV-EDVI >95 ml/m^2^ for females, >100 ml/m^2^ for males; RV-EDVI >96 ml/m^2^ for females, >111 ml/m^2^ for males. The ventricular function was classified as reduced, if the ejection fraction was below the lower limit calculated as mean -2SD of the normal collective considering gender and age <60 years: LV-EF <58 % for females, <57 % for males; RV-EF <52 % for females, <50 % for males. LV-MM was classified as increased, if LV-MMI was above the upper limit determined as mean +2SD of the normal collective considering gender and age <60 years: LV-MMI >77 g/m^2^ for females, >91 g/m^2^ for males.

Additionally, the right ventricular diameter was measured in four-chamber view 4CV. RV dilatation was assumed if cross-section dimension in 4CV and basal short-axis was above two standard deviations of the normal value according to Hergan et al. [16].

LV myocardial thickness was evaluated in a mid-ventricular short-axis slice in segment 9 (inferoseptal) and 11 (inferolateral) and rated according to Kawel et al. [17]: LV myocardium was considered hypertrophic in segment 9 exceeding 9.3 mm (women) and 11.4 mm (men), respectively; in segment 11 exceeding 7.5 mm (women) and 9.1 mm (men).

RV myocardial thickness was measured in the posterior wall in a mid-ventricular short-axis slice. RV hypertrophy was assumed if RV wall thickness was 5 mm or higher [18, 19]. Maximum thickness of pericardial effusion was measured in end-diastole in Steady State Free Precession (SSFP) cine loops. Pericardial effusion was classified minimal ≤5 mm pericardial space, and moderate >5 mm [20].

#### Late Gadolinium enhancement and inversion time localizer

Inversion time localizer nulling patterns of the myocardium and blood pool were categorized by the nulling type patterns 1 (normal) and the abnormal patterns 2–4 as imposed by Pandey et al. [21]: Physiologic type 1 nulling pattern: blood pool nulls first, followed by myocardium and spleen at the same time. Pathologic type 2 nulling pattern: myocardium nulls first, followed by blood pool and spleen at the same time. Pathologic type 3 nulling pattern: myocardium and blood pool null simultaneously, followed by the spleen. Pathologic Type 4 nulling pattern: myocardium, blood pool and spleen all null at different times.

LGE was evaluated qualitatively and semi-quantitatively. Datasets in which the Inversion Time (TI) Scout indicated an insufficient nulling of the myocardium in contrast to the blood pool were excluded from LGE quantification. For quantification of LGE contours in all short axis slices were drawn manually with care to exclude pericardial fat and blood pool at the myocardial borders. For visual reading and semi-automated thresholding, the recommendations of the Society for Cardiovascular Magnetic Resonance Task Force were adopted [22]: signal intensity >5 standard deviations of the normal myocardium was considered as full intensity LGE. In addition, a grayscale analysis of intermediate-signal intensity LGE was performed (≥2SD but <5SD of the normal myocardium). Full intensity and intermediate intensity LGE were recorded in % of the myocardial mass. Inversion time for the first and last LGE image was recorded.

### Statistical analysis

Statistical analysis was performed using JMP (Version 12.2, SAS Institute Inc., Cary NC, USA). All continuous variables are expressed as mean value ± standard deviation and range in square brackets. Interobserver-variability for continuous variables was evaluated with Bland-Altman difference plots. Mean difference and the limits of agreement defined as ±1.96 SD were calculated for LV-EDV, LV-EF, RV-EDV and RV-EF. Over all categorical variables in visual analysis interobserver-agreement was measured with Cohen’s Kappa. Paired two-sided t-test was applied for null hypothesis if Kappa equals zero. Agreement in Kappa statistic was rated according to Landis and Koch [23]: ‘moderate’ (0.41–0.6), ‘substantial’ (0.61–0.8), and ‘almost perfect’ (0.81–1).

To test if among the study cohort more than the 2.5 % of the normal collective had pathologic values, 95 % confidence interval (CI) was calculated for the quotient *pathologic sample/total sample* for LV-EF, RV-EF, LV-EDVI, and RV-EDVI. Higher occurrence of pathologic values in the study cohort was assumed if lower 95 % CI was above 2.5 %. Normal distribution for each value was assessed visually in curves.

To determine how many pathologic findings occurred in one patient, CMR findings were categorized: 1) moderate pericardial effusion; 2) ventricular kinesic pattern (LV and RV hypokinesia, dyskinesia, dyssynchrony, and diastolic restriction); 3) reduced ventricular function (reduced LV-EF or LV-EF); 4) suspect for fibrosis and inflammation (positive LGE); 5) right ventricular dilatation.

## Results

### Endomyocardial biopsy results

Myocardial fibrosis was found in all patients. The mean degree of myocardial fibrosis over all patients was 12.3 % (±6.3 %; [4 %; 32 %]). Five patients (25 %) had an inflammation grade 1; ten patients (50 %) grade 2; five patients (25 %) grade 3. One patient had 300 copies/μg myocardial DNA of parvovirus B19 genotype 1, indicating virus persistence.

### Morphology and function

In visual motion analysis, septal regional LV hypokinesia was found in 15 patients (75 %); LV intraventricular dyssynchrony in 13 patients (65 %); and LV diastolic dysfunction of a restrictive pattern with passive filling in 17 patients (85 %). Regional or global RV hypokinesia was found in 14 patients (70 %); RV dyskinesia in 2 patients (10 %); RV intraventricular dyssynchrony in 12 patients (60 %); and restrictive RV diastolic dysfunction in 13 patients (65 %). 19 patients (95 %) had pathologic LV and or RV ventricular kinesia pattern (CMR cine loops see Additional file 1 and Additional file 2).


**Fig. 1:**
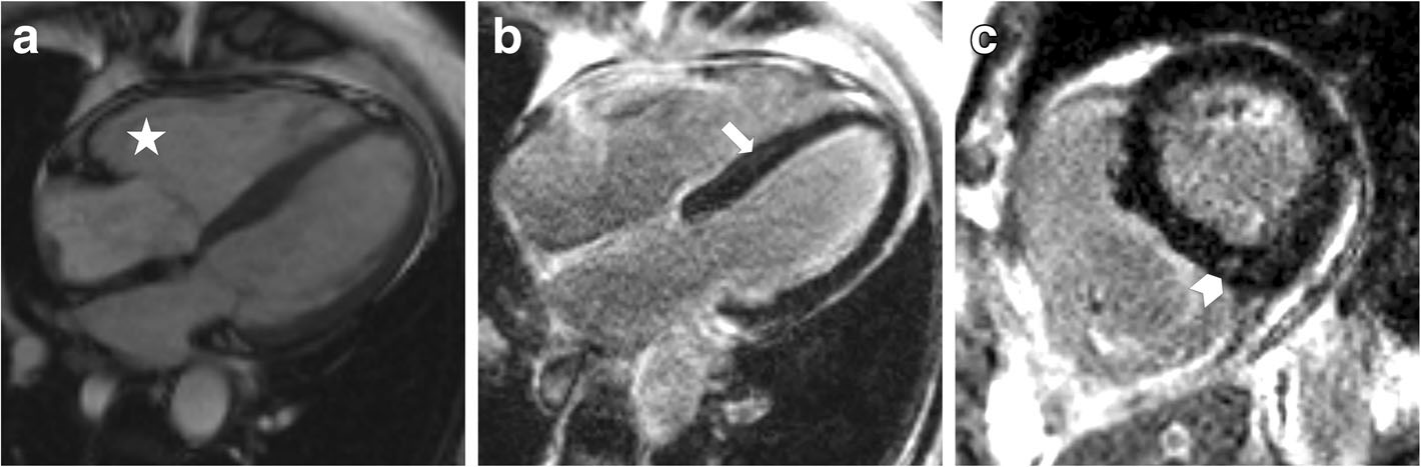
Depicts typical, subtle findings in myocardial involvement in SSc in a female patient: **a** Right ventricular dilatation in SSFP 4CV (star). Cine loop is available in Additional file 1 to depict characteristically left ventricular and right ventricular restrictive filling pattern. LGE images in 4CV (**b**), and mid-ventricular SA (**c**) depict mid-myocyardial intermediate-signal intensity linear (*arrow*) and patchy (*arrowhead*) LGE. *4CV: Four-chamber view; LGE: Late Gadolinium Enhancement; SA: Short-axis; SSFP: Steady State Free Precession*

Additional file 1: SSFP loop of Fig. 1a in a female patient depicts characteristically left ventricular and right ventricular restrictive filling pattern in cardiac involvement in SSc. *Systemic Sclerosis.*

Additional file 2: The SSFP loop depicts minimal pericardial effusion and right ventricular dilatation in a male patient in four-chamber view SSFP. The loop demonstrates intraventricular and interventricular dyssynchrony as well as restriction in diastole. *SSFP: Steady State Free Precession.*



The volumetric analysis indicated left ventricular dilatation in 4 patients (20 %; 95%CI: 8–41 %) and right ventricular dilatation in 6 patients (30 %; 95%CI: 15–54 %). Ventricular function was reduced in 14 patients: 14 patients (70 %; 95%CI: 51–88 %) had reduced RV-EF; 10 of which (50 %; 95%CI: 30–70 %) had both reduced LV-EF and RV-EF. Increased LV-MMI was found in two patients (10 %) by left ventricular myocardial mass volumetry (Table 1). The mean right ventricular diameter was 48.7 mm (±7 mm; [35 mm; 59 mm]). 16 patients (80 %) had RV diameters exceeding the upper limit for normal RV diameter (Fig. 1).

**Table 1:** Continuous variable data

	Absolute value male and female	Normalized to BSA
LV-EF	57 % (±7 %; [39 %; 70 %])	
LV-EDV	137 ml (±43 ml; [80 ml; 210 ml])	73 ml/m^2^ (±21 ml/m^2^; [38 ml/m^2^; 105 ml/m^2^])
LV-MM	124 g (±31 g; [79 g; 205 g])	65 g/m^2^ (±17 g/m^2^; [26 g/m^2^; 111 g/m^2^])
RV-EF	45 % (±7 %; [28 %; 57 %])	
RV-EDV	167 ml (±43 ml; [105 ml; 242 ml])	89 ml/m^2^ (±20 ml/m^2^; [50 ml/m^2^; 117 ml/m^2^])

*BSA Body Surface area in* *m* ^*2*^, *LV-EF Left ventricular ejection fraction*, *LV-EDV Left ventricular end-diastolic volume*, *LV-MM Left ventricular myocardial mass*, *RV-EDV Right ventricular end-diastolic volume*, *RV-EF Right ventricular ejection fraction*

End-diastolic myocardial thickness of the LV in the mid-ventricular septum (segment 9) was 10.4 mm (±1.8 mm; [8 mm; 14 mm]), in the inferolateral wall ﻿(segment 11) 8.1 mm (±1.8 mm; [5 mm; 11 mm]). 7 patients (35 %) had septal myocardial hypertrophy (segment 9), 7 patients (35 %) had inferolateral myocardial hypertrophy (segment 11). Five of these patients had septal and inferolateral myocardial hypertrophy, 2 patients had asymmetric septal hypertrophy.

RV Myocardial thickness was 3.3 mm (±1.2 mm; [1 mm; 5 mm]), normal 1–4 mm in 18 patients (90 %), and pathologic 5 mm in two patients (10 %).

**Fig. 2:**
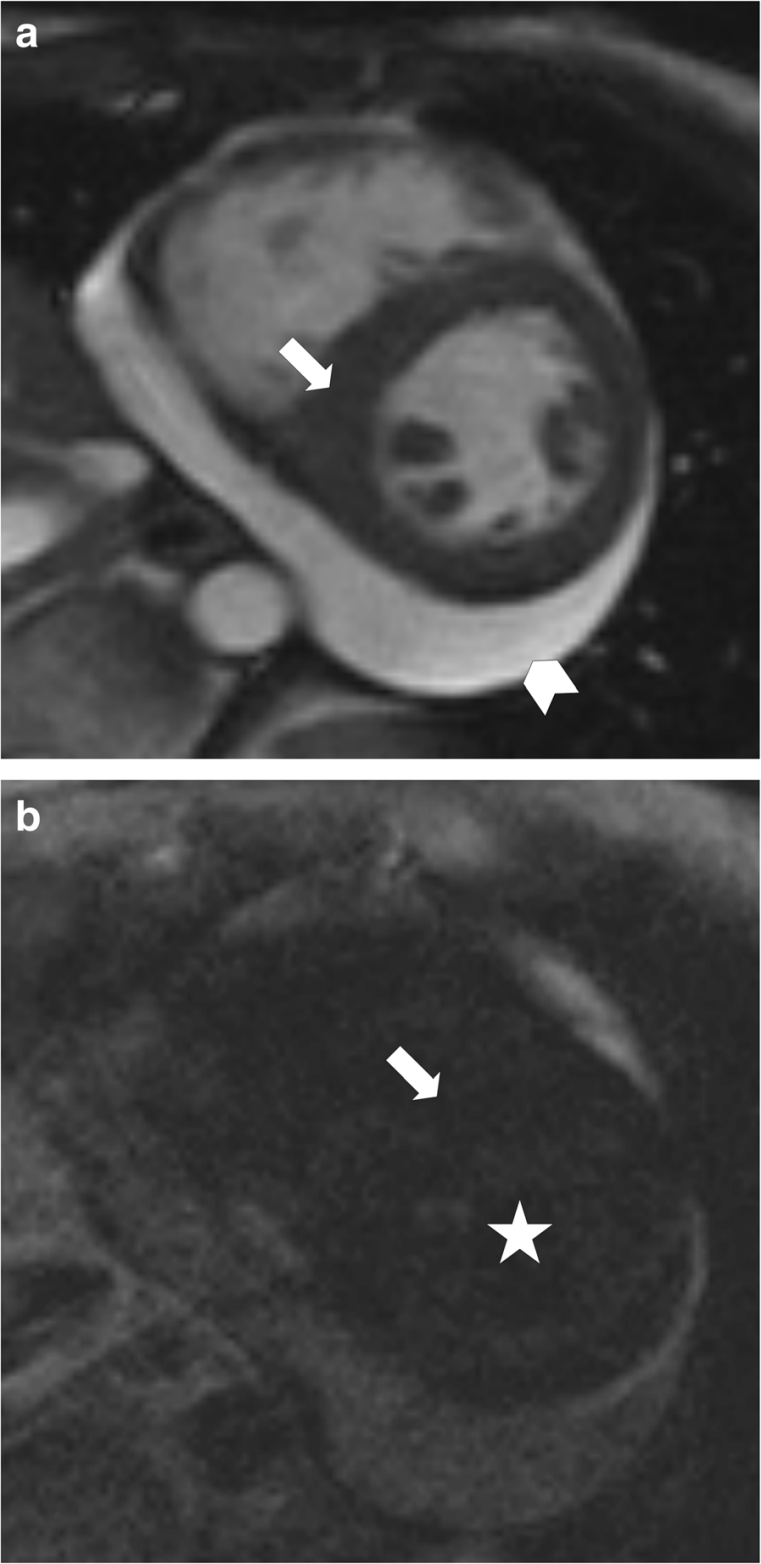
Depicts septal hypertrophy (*arrow*) and moderate pericardial effusion (*arrowhead*) in a female patient in mid-ventricular short-axis SSFP imaging (**a**). In LGE imaging (**b**) of the same slice no sufficient nulling of the myocardium (*arrow*) in contrast to the blood pool (*star*) was possible due to diffuse LGE in diffuse fibrosis without remote and healthy myocardium. The finding may be misinterpreted as technical failure. *LGE: Late Gadolinium Enhancement; SSFP: Steady State Free Precession*

Seven patients (35 %) had minimal pericardial effusion, nine patients (45 %) had moderate pericardial effusion >5 mm (Fig. 2 and Additional file 1 and Additional file 2).

### Inversion time localizer

**Fig. 3:**
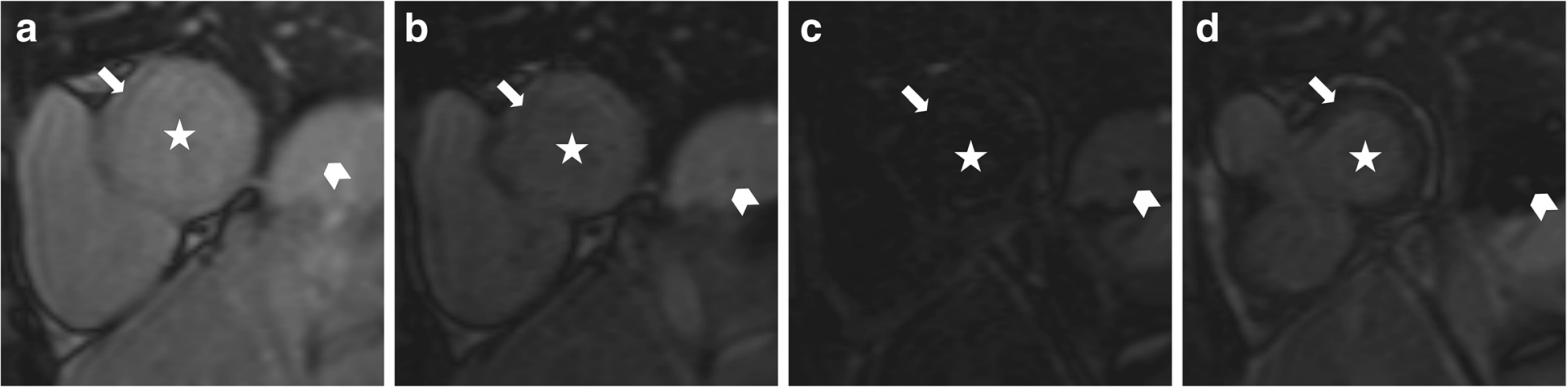
Depicts pathologic type 3 nulling pattern of myocardium in inversion time localizer in a male patient according to Pandey et. al. [21]. Images depict a basal short-axis slice with different inversion times (TI): **a** TI 80 ms; **b** TI 160 ms; **c** TI 240 ms; **d** TI 320 ms. Myocardium (*arrow*) and blood pool (star) null simultaneously, followed by the spleen (*arrowhead*). This phenomenon indicates diffuse gadolinium enhancement in the myocardium and leads to insufficient contrast in LGE imaging as shown in Fig. 2. *LGE: Late Gadolinium Enhancement*

Inversion time localizer was not evaluable in three patients. Physiologic type 1 nulling pattern was found in eight of the evaluable 17 patients (47 %). Pathologic nulling patterns were found in nine patients (53 %): Type 2 in two patients (12 %); type 3 in five patients (29 %, Fig. 3), type 4 in two patients (12 %).

### Late Gadolinium Enhancement

LGE imaging was not evaluable in one patient due to breathing artifacts. In the visual analysis, three of 19 patients (16 %) had intermediate signal-intensity LGE in linear and patchy pattern (Fig. 1). Ten patients (53 %) had diffuse LGE. Two of these patients had generalized LGE with no reasonable nulling of the myocardium (Figs. 2 and 3), they were excluded from semi-quantitative analysis.

In the semi-quantitative analysis, pathologic LGE was found in all patients: full intensity LGE was found in 5.1 % (±3.4 %; [0 %; 11 %]) of the LV myocardial mass in patchy and linear patterns. Intermediate-signal intensity LGE was found in 25.9 % (±5.7 %; [17 %; 37 %]) of the LV myocardial mass.

Inversion time for LGE imaging has been set to 283 ms (±25 ms; [230 ms; 320 ms]) for the first image, for the last image to 312 ms (±34 ms; [260 ms; 380 ms]). TI was elevated from first to last LGE image for 28 ms (±23 ms; [0 ms; 80 ms]).

All results are summarized by frequency in Table 2.

**Table 2:** Frequency of abnormal CMR findings

Pathology	Frequency	Appearance	Normal value
pathologic LV and or RV ventricular kinesic pattern	95 %		-
*LV diastolic dysfunction*	*85 %*		-
*LV hypokinesia*	*75 %*		-
*RV hypokinesia*	*70 %*		-
*RV diastolic dysfunction*	*65 %*		-
*LV intraventricular dyssynchrony*	*65 %*		-
*RV intraventricular dyssynchrony*	*60 %*		-
*RV dyskinesia*	*10 %*		-
Pericardial Effusion	80 %		
*Minimal Effusion*	*35 %*	≤5 mm	
*Moderate Effusion*	*45 %*	>5 mm	≤5 mm [20]
RV-EF	74 %	below -2SD of normal	≥52 % (f); ≥50 % (m) ; <60 years [15]
LV-EF	50 %	below -2SD of normal	≥58 % (f); ≥57 % (m) ; <60 years [15]
RV-EDVI	32 %	above +2SD of normal	≤96 ml/m^2^ (f); ≤111 ml/m^2^ (m) ; <60 years [15]
LV-EDVI	20 %	above +2SD of normal	≤95 ml/m^2^ (f); ≤100 ml/m^2^ (m) ; <60 years [15]
LV-MMI	10 %	above +2SD of normal	≤77 g/m^2^ (f); ≤91 g/m^2^ (m); <60 years [15]
RV EDD in 4CV	80 %	above +2SD of normal	<39 mm (f); <49 mm (m) [16]
LV myocardial thickness	35 %	above +2SD of normal	Segment 9: ≤9.3 mm (f); ≤11.4 mm (m) Segment 11: ≤7.5 mm (f); ≤9.1 mm (m) [17]
RV myocardial thickness	10 %	≥5 mm	<5 mm [18, 19]
Inversion time localizer	50 %	Pathologic nulling pattern	Blood nulls first, myocardium and spleen second [21]
LGE visual analysis	69 %		-
*Visual diffuse*	*53 %*	diffuse, or no reasonable nulling	-
*intermediate-signal intensity*	*16 %*	linear and patchy	-
LGE semi-quantitative analysis	100 %		
*intermediate-signal intensity*	*100 %*	17–37 % of LV mass	-
*full intensity*	*94 %*	0–11 % of LV mass	-

*4CV Four-chamber view, EDD End-diastolic diameter, LGE Late Gadolinium enhancement, LV left ventricle, LV-EDVI Left ventricular end-diastolic volume index, LV-EF Left ventricular ejection fraction, LV-MMI Left ventricular myocardial mass index, RV right ventricle, RV-EDVI Right ventricular end-diastolic volume index, RV-EF Right ventricular ejection fraction, SD Standard deviation*

In evaluation of the initial clinical CMR reports blinded to subsequent EMB, the report was diagnosing ‘cardiac SSc involvement’ in 6 patients (30 %).

### Statistical analysis

The interobserver variability analysis indicated a mean difference for LV-EDV of -1.8 ml with limits of agreement -11.4 ml; 7.9 ml. The mean difference for LV-EF was 0.7 %, limits of agreement were -5.5 %; 7 %. The mean difference for RV-EDV was 1.7 ml, limits of agreement were -7.9 ml; 11.4 ml. The mean difference for RV-EF was 0.2 %, limits of agreement were -5.7 %; 6.1 %. Kappa statistic for interobserver-agreement in categorical variables was ‘almost perfect’ 0.913, p < 0.0001. LV-EF, RV-EF, LV-EDVI, and RV-EDVI were normally distributed.

In categorical analysis of pathologic CMR findings, seven patients (35 %) had positive findings in three categories, nine patients (45 %) in four categories, and four patients (20 %) in five categories.

## Discussion

In the present study we systematically evaluated CMR findings in a rare subgroup of SSc patients with histopathologically confirmed myocardial involvement.

In CMR, pathologic findings were documented in all SSc patients, confirming a high occurrence of abnormal volumetric data as compared to normal healthy collectives (95%CI). Diastolic dysfunction is known to develop early in the course of myocardial involvement in SSc even before the appearance of clinical symptoms [24]. The pericardial effusion found in the majority of our patients is known to be common in SSc [25], and might contribute to the development of diastolic dysfunction [26]. Pericardial effusion is also common in pulmonary arterial hypertension [25], reflecting the complexity of cardiopulmonary involvement in SSc. Notably, a number of patients only had a slight or minimal pericardial effusion, that will commonly be classified as unspecific. Regional or global enhancement by LGE imaging indicating myocardial fibrosis can be regarded as morphologic correlate for restricted diastolic and systolic function as well as for myocardial electrical conduction disorders, potentially resulting in ventricular dyssynchrony. RV dilatation occurred independently from pulmonary hypertension in a relevant number of patients, a phenomenon that has already been described previously [9]. In this context, RV dilatation may indicate RV involvement as a primary symptom in contrast to secondary dilatation.

In visual reading and semi-quantitative evaluation, we found typical non-coronary distribution of full intensity LGE as described before: a linear enhancement [7]; as well as patchy pattern at the insertion points of the right ventricle—which is also typically found in hypertrophy and pulmonary artery hypertension, the latter being frequently co-existent due to severe lung involvement in SSc patients [27]. In the semi-quantitative evaluation, grayscale intermediate intensity LGE was most frequent. We performed semi-quantitative T_1_ evaluation with an ordinal scale by means of the inversion time localizer. Pathologic nulling patterns of inversion time localizer were common in our patient group, indicating severe diffuse myocardial fibrosis. Diffuse myocardial fibrosis frequently evades visual detection and is significantly underestimated in LGE quantification, for nulling of the myocardium suppresses general enhancement by shifting the threshold [28]. As a result, diffusely involved myocardium may appear normal. In semi-quantitative LGE evaluation, the region of interest for remote myocardium may be placed in regions with less but still pathologic contrast enhancement, visually not identified as such. Although diffuse general fibrosis can be detected and quantified using T_1_-mapping, the values remain unspecific for diffuse fibrosis [29]. We did not find a ‘runaway’ TI with initially short TI and large incremental steps during image acquisition, which is known to be typical for amyloidosis [30]. In consequence, non-invasive assessment of the extent of myocardial fibrosis in SSc remains challenging and the implication of imaging findings for prognosis and further treatment remains uncertain. Fibrosis in biopsy may only correlate with T_1_- mapping for absolute quantification of fibrosis. None of our parameters is likely to correlate with fibrosis in biopsy: LGE does shift threshold of healthy towards pathologic when nulling myocardium, and functional parameters are subject to situational adaption. Extent of cardiac involvement in SSc is not necessarily dependent on other objectively verifiable parameters and subject to subclinical periods, primary or secondary involvement and influenced by cardiotoxic therapy [2, 12].

CMR is accepted as one of the non-invasive modalities to investigate SSc involvement of the heart [31]. Yet, to determine the degree of cardiac involvement seems to be difficult even for experienced readers. To date, criteria for the CMR diagnosis of myocardial involvement in SSc are not defined. CMR was performed and evaluated before EMB in all patients, so that no influence on imaging and initial blinded reading can be stated.

As systolic dysfunction is a rare finding in SSc, the severity of the involvement tends to be underestimated [32]. Based on the inversion time localizer principle, T_1_-mapping should be a promising method to further investigate diffuse myocardial fibrosis in SSc involvement [29]. T1-mapping does not require healthy remote myocardium to detect diffuse fibrosis. Extracellular volume determination in T1-mapping has recently shown the potential to correlate with myocardial fibrosis [33].

Our results might contribute to improve the non-invasive diagnosis of cardiac involvement in SSc, using CMR to identify and estimate cardiac involvement. Discrete CMR imaging patterns could be found concurrently, also in severe SSc myocardial involvement. In our retrospective evaluation, myocardial involvement in SSc was systematically underdiagnosed in the initial clinical report blinded to the subsequent histopathologic work-up with true positive diagnoses in only 30 % of our SSc cohort. The main reason may be underestimation of subtle morphologic and functional findings in SSc involvement, as this is a rare cardiac diagnosis. To date, mainly CMR findings in the maximum degree of occurrence have been published, potentially leading to a trend to underestimate subtle, yet SSc-typical, findings. Most CMR findings appear to be unspecific and can be found in a variety of diseases. However, the combination of certain findings as described in our work seems to be characteristic for cardiac SSc involvement. For the lack of correlation, CMR cannot replace EMB. EMB itself is rarely performed in a clinical setting. Application of the proposed criteria may improve assessment of clinically relevant SSc involvement. Although CMR findings in patients with cardiac involvement in SSc can easily go underdiagnosed and classified as unspecific, the combination of several CMR findings is frequent and therefore allows the diagnosis of cardiac involvement in patients with SSc by CMR: Besides distinct LGE patterns, most important findings are involvement of both ventricles, pathologic LV/RV contractility, dyssynchrony or restriction. Reading of inversion time localizer for diffuse LGE and can be easily implemented without additional technical requirements.

This approach might be useful for risk stratification when considering therapeutic options, and may eventually reduce morbidity and mortality in a patient group at risk for major adverse cardiac events. Currently two studies are recruiting SSc patients eligible for autologous stem cell transplantation, one in Chicago, IL, USA and one in Tübingen, Germany (ClinicalTrials.gov NCT01445821; NCT01895244) [34]. These studies aim to reduce cardiac toxicity of the therapy regimen with effective use of stem cell transplantation [11].

### Study limitations

The study population is comparably small and the study CMR image readers were biased by knowledge of histopathologically proven involvement in SSc in all patients. Only patients with severe disease were examined. Mild and limited cutaneous SSc might present with less pathologic CMR findings per individual. No T_2_ imaging was performed, but non-quantitative T_2_ imaging cannot reliably depict diffuse edema [35, 36] and edema has not been found in previous reports [10]. Neither T_1_ nor T_2_- mapping were available at the time patients were examined.

## Conclusions

CMR seems to be a valuable tool to identify and assess the extent of cardiac involvement and therefore might be useful for risk stratification and making a decision upon therapeutic strategies. Myocardial involvement in systemic sclerosis can be assumed if pathologic findings in three categories are found: 1) pericardial effusion; 2) ventricular kinesic pattern (LV and RV hypokinesia, dyskinesia, dyssynchrony, and diastolic restriction); 3) reduced LV-EF or RV-EF; 4) positive LGE or pathologic inversion time localizer; 5) right ventricular dilatation.

## Abbreviations

4CV: Four-Chamber View
BSA: Body Surface Area
CI: Confidence Interval
CMR: cardiovascular magnetic resonance
dcSSc: diffuse cutaneous SSc
EMB: Endomyocardial Biopsy
lcSSc: limited cutaneous SSc
LGE: Late Gadolinium Enhancement
LV: Left Ventricle; Left Ventricular
LV-EDV: Left Ventricular Enddiastolic Volume
LV-EDVI: Left Ventricular Enddiastolic Volume Index
LV-EF: Left Ventricular Ejection Fraction
LV-MM: Left Ventricular Myocardial Mass
LV-MMI: Left Ventricular Myocardial Mass Index
RV: Right Ventricle; Right ventricular
RV-EDV: Right Ventricular Enddiastolic Volume
RV-EDVI: Right Ventricular Enddiastolic Volume Index
RV-EF: Right Ventricular Ejection Fraction
SA: Short-Axis
SD: Standard Deviation
SSc: Systemic Sclerosis
SSFP: Steady State Free Precession
TI: Inversion Time
